# Development of an Artificial Intelligence-Based System for Predicting Fall Risk in Neurological Patients

**DOI:** 10.17691/stm2026.18.4.05

**Published:** 2026-08-31

**Authors:** E.S. Ikonnikova, A.E. Slotina, G.A. Belyankin, M.A. Zabello, V.Yu. Vishnyakov, A.V. Lobanov, N.V. Bedeneu, A.A. Dobrovolsky, D.O. Zemlyanaya, A.Yu. Tropynina, O.A. Kirichenko, A.V. Baidukova, N.S. Suponeva

**Affiliations:** Junior Researcher, Laboratory of Neurointerfaces, Institute of Medical Rehabilitation and Restorative Technologies; Russian Center of Neurology and Neurosciences, 80 Volokolamskoe Shosse, Moscow, 125367, Russia; MD, PhD, Researcher, Institute of Medical Rehabilitation and Restorative Technologies; Russian Center of Neurology and Neurosciences, 80 Volokolamskoe Shosse, Moscow, 125367, Russia; PhD, Associate Professor, Department of Operations Research, Faculty of Computational Mathematics and Cybernetics; Lomonosov Moscow State University, 1 Leninskie Gory, Moscow, 119991, Russia; Master, Department of Operations Research, Faculty of Computational Mathematics and Cybernetics; Lomonosov Moscow State University, 1 Leninskie Gory, Moscow, 119991, Russia; Cofounder; LLC Shagometrika, 19, Bldg 2, office 3P, Sadovaya-Spasskaya St., Moscow, 107078, Russia; Cofounder; LLC Shagometrika, 19, Bldg 2, office 3P, Sadovaya-Spasskaya St., Moscow, 107078, Russia; Resident Physician; Russian Center of Neurology and Neurosciences, 80 Volokolamskoe Shosse, Moscow, 125367, Russia; Resident Physician; Russian Center of Neurology and Neurosciences, 80 Volokolamskoe Shosse, Moscow, 125367, Russia; Resident Physician; Russian Center of Neurology and Neurosciences, 80 Volokolamskoe Shosse, Moscow, 125367, Russia; Resident Physician; Russian Center of Neurology and Neurosciences, 80 Volokolamskoe Shosse, Moscow, 125367, Russia; Head of Rehabilitation Department; Russian Center of Neurology and Neurosciences, 80 Volokolamskoe Shosse, Moscow, 125367, Russia; Resident Physician; Russian Center of Neurology and Neurosciences, 80 Volokolamskoe Shosse, Moscow, 125367, Russia; MD, DSc, Professor, Corresponding Member of Russian Academy of Sciences, Director of Institute of Medical Rehabilitation and Restorative Technologies; Russian Center of Neurology and Neurosciences, 80 Volokolamskoe Shosse, Moscow, 125367, Russia

**Keywords:** fall risk diagnostics, gait disorders, balance disorders, movement biomechanics, artificial intelligence

## Abstract

**The aim of the study** was to develop an artificial intelligence model for predicting the probability of falls based on the analysis of video recordings of gait patterns.

**Materials and Methods:**

The study involved 187 patients (median age 59 years) with neurological diseases of various etiologies who complained of impaired balance and unsteadiness while walking. All participants underwent video recording of their movements using a smartphone during a 10-meter walk test and Timed Up and Go test. Based on the analysis of 2077 steps recorded in 122 patients, an algorithm for fall risk stratification was developed using the YOLO-NAS Pose M architecture and a two-layer machine learning model.

**Results:**

The final model when analyzing video data achieved 76% accuracy in risk prediction, with an average absolute error of time parameter prediction of 1.445 s. The following parameters were identified as key biomechanical predictors of fall risk: step base width at the ankle joint level, lateral trunk sway, step base width at the knee joint level, vertical foot clearance during the swing phase, and temporal gait characteristics.

## Introduction

According to the World Health Organization, there are 37.3 million falls globally each year that require medical attention. Research shows that the highest risk of falls is among the elderly (aged 60–80) [1], with this issue affecting up to 50% of people over the age of 80 [2–4]. Approximately 20–30% of patients who experienced a fall in older age require emergency medical care and various costly surgeries [2, 5, 6].

Neurological disorders significantly contribute to the risk of falls and balance disturbances [7, 8]. Retrospective studies of patient histories indicate falls to be most frequently observed in neurological patients in emergency settings, and they are influenced by age and the duration of hospitalization [9]. It is worth noting that most falling neurological patients are elderly. A systematic review [10] indicates that among younger patients with neurological disorders, when controlling for age, the proportion of individuals who have experienced at least one fall episode reaches 50%. This fact points to an independent contribution of neurogenic mechanisms to the overall fall risk. Expert estimates suggest that the costs of treating fall-related injuries (e.g., hip fractures) in the healthcare system of the Russian Federation can reach 300,000 rubles, with the subsequent long-term rehabilitation costing up to 1 million rubles. Therefore, reducing the number of falls among patients with balance disorders represents a socially significant challenge. The issue of relevance will steadily increase as the age of active longevity rises.

In terms of neurological symptoms most closely associated with fall risk, the leading ones include cognitive impairments, disturbances in superficial and deep sensitivity, and motor function disorders and impaired mobility [11]. When analyzing the main causes of falls in patients, accidental (sudden) falls related to the environment typically rank first — 31%, followed by the falls associated with gait and balance disturbances — 17% [12].

One of the main solutions to the problem of falls in patients with neurological conditions is timely and accurate risk assessment followed by preventive measures. Two approaches are used for such assessment: clinical and instrumental, with the number of tools employed, according to meta-analyses, exceeding 20 [13]. The clinical approach relies on the use of diagnostic clinical scales, tests, and questionnaires. Brief screening methods typically involve asking the patient about key risk factors for falls (history of falls, medication use, the presence of intravenous drug delivery systems, comorbidities, etc.) and a qualitative subjective assessment of 1–2 motor impairments (walking, mobility, unsteadiness, etc.) [14]. This approach has its drawbacks; however, it addresses the task of basic mass screening [15] in inpatient facilities [16, 17].

A more detailed and specific clinical assessment involves the use of tests and scales evaluating fall risk associated with an impaired motor function: Timed Up and Go (TUG) test, Berg Balance Scale, Tinetti Scale, mini-BEST test, and others. It is important to note that there is no universal approach to determine fall risk using these scales: expert opinions vary, complicating a comprehensive assessment of publications in this area. For neurological patients, Berg Balance Scale and the TUG test are the most sensitive and specific for assessing fall risks [13]. These tools are more convenient and applicable compared to Tinetti Scale and Dynamic Gait Index (which are descriptive) and require no additional equipment (inclined surfaces, etc.) like Fullerton Advanced Balance Scale or mini-BEST test.

Despite the widespread use of clinical scales for assessing fall risk in neurological patients, they all share one significant drawback — subjectivity [13], which means they are operator-dependent. Objective and more accurate evaluations of balance impairments in neurological diseases are provided by biomechanical assessment methods: computerized stabilometry and gait analysis. Obviously, objective instrumental methods (stabilographic platforms, motion capture systems, stereo cameras, special markers, wearable sensors) require the testing specialist to have skills in conducting the research and interpreting the data.

In recent years, automated computer solutions have been actively developed [18–21]. The most complex task — gait analysis — has been entrusted to artificial intelligence (AI): currently, there are dozens of studies in the field of diagnosing various diseases based on gait (Parkinson’s disease, depression, and many others) [22–24] including those in the early stages. Determining fall risk is a less complex and specific task, making it potentially feasible when processing video recordings with an AI system. Today, AI image processing from stereo cameras and sensors is successfully implemented, and the so-called human pose estimation (HPE) models, which assess human posture and movements from 2D video captured by a single camera, have significantly improved [25].

**The aim of the study** was to develop a diagnostic model of balance disorders and fall risk in neurologic patients based on AI analysis of video recordings.

## Materials and Methods

The study involved 187 (87 male and 100 female) patients with neurologic disorders of various etiology (Table 1), complaining of instability and shakiness when walking. The median age was 59 [46; 69] years.

**Table 1:** Distribution of patients by nosologies (according to ICD-10)

ICD-10 code	Diseases according to ICD-10	Number of patients
I69.3	Cerebral infarction sequelae	49
I67.8	Other specified cerebral vascular injuries	25
G35	Multiple sclerosis	18
G12.2	Motor neuron disease	15
G20	Parkinson’s disease	14
G31.9	Degenerative disease of the nervous system, non-specified	9
G11.2	Late-onset cerebellar ataxia	6
I69.1	Intracranial hemorrhage sequelae	5
G31.8	Other specified degenerative diseases of the nervous system	4
G40.2	Localized (focal) (partial) symptomatic epilepsy and epileptic syndromes with complex partial spasmodic seizures	3
I67.3	Progressive vascular leukoencephalopathy	3
E83.0	Copper metabolism disorder	2
G04.8	Other encephalitis, myelitis, and encephalomyelitis	2
G11.4	Hereditary spastic paraplegia	2
G23.1	Progressive supranuclear ophthalmoplegia [Steele–Richardson–Olszewski syndrome]	2
G37.9	Demyelinating disease of the central nervous system non-specified	2
G62.9	Polyneuropathy non-specified	2
G71.0	Muscular dystrophy	2
D33.0	Benign disease of the brain and other central nervous system parts	1
G10	Huntington’s disease	1
G23.2	Multiple system atrophy, Parkinsonian type [MSA-P]	1
G23.3	Multiple system atrophy, cerebellar type [MSA-C]	1
G25.8	Other specified extrapyramidal and motor impairments	1
G36.0	Neuromyelitis optica [Devic disease]	1
G36.9	Acute disseminated demyelination, non-specified	1
G37.5	Concentric sclerosis [Balo’s disease]	1
G37.8	Other specified demyelinating diseases of the nervous system	1
G40.9	Epilepsy non-specified	1
G54.5	Neuralgic amyotrophy	1
G54.8	Other nerve root and plexus disorders	1
G61.0	Guillain–Barre syndrome	1
G72.4	Inflammatory myopathy, not elsewhere classified	1
G90.8	Other disorders of the autonomic nervous system	1
G95.1	Vascular myelopathies	1
G95.8	Other specified spinal diseases	1
I62.9	Intracranial hemorrhage (nontraumatic) non-specified	1
I69.0	Subarachnoid hemorrhage sequelae	1
M48.0	Spinal stenosis	1
M54.6	Pain in the thoracic spine	1
T90.5	Intracranial injury sequelae	1

27 patients had the history of diagnosed locomotor disorders: surgical decompression of the spinal canal at lumbar level (n=8), compression fracture of thoracic vertebrae (n=2), osteoarthrites of the lower limb joints (n=11), arthrites (n=2), lower limb injuries older than 1 year and less than 3 years (n=4); 4 patients required joint replacement of the lower limbs.

100 patients had diagnosed vision disorders: amblyopia (n=3), hemianopsia (n=9), glaucoma (n=2), cataract (n=39), macular degeneration (n=1), myopia and astigmatism (n=39), partial optic nerve atrophy (n=6), uveitis (n=1).

The study was conducted in accordance with the Helsinki Declaration (2024) and approved by the local Ethics Committee of the Russian Center for Neurology and Neuroscience (protocol No.9-4/25 dated October 20, 2025). All patients provided informed consent.

Before starting the video recording of the walks, all patients were assessed using TUG test to determine their fall risk group [26].

The 10-meter walk test was conducted by patients in comfortable footwear at a comfortable speed; in footwear at maximum speed; barefoot at a comfortable speed; and barefoot at maximum speed.

The walking was recorded on two smartphones positioned 14 meters away. The patient began at the start of a non-slip 10-meter track, and their task was to walk to the end of the track at either a comfortable or maximum speed. For video analysis, the first and last 2 m were excluded (acceleration and deceleration phases) — similar to the modified 10-meter walk test [27].

The TUG test was also recorded using two smartphones placed 4 meters away from each other. Thus, a total of 10 video recordings of walking were obtained for each patient, which were later used to create a fall risk diagnostic model using AI. The initial dataset for training AI model included gait parameters from 148 patients (1,285 video recordings and 8,329 steps, identified by the moments of right foot lift-off). During the data preprocessing stage, we excluded the patients who used assistive supports — crutches or canes (a total of 8 patients, corresponding to 55 video recordings and 510 steps). The training sample was formed using the video recordings in which patients moved away from the camera. This sample included 140 patients, 612 video recordings, and 3,972 steps.

The video images were compiled into a single anonymized database. The subsequent processing and analysis were carried out as follows. Initially, the data were manually cleaned at the individual step level. Steps were excluded if there were any of the following criteria:

1. if the discrepancy between the actual step length and the length predicted by Fourier method exceeded 20%;
2. if the relative duration of the stance phase fell outside the range of 0.3–0.7 of the actual step length;
3. if the minimum angle of hip flexion was outside the range of 50–90°;
4. if the minimum amplitude of ankle movement exceeded 2°;
5. if the maximum deviation of the ankle along the X-axis was less than 0.35;
6. if the maximum deviation of the shoulder girdle along the X-axis was outside the range of 0.7–0.86;
7. if the minimum deviation of the ankle along the X-axis was less than 0.

After applying all the listed criteria, the final training dataset included the data from 122 patients (411 video recordings and 2,077 steps).

To create a model to diagnose the risk of falling using AI, this work examined four neural network architectures for pose estimation. One of the pose estimation algorithms used was the OpenPose architecture. In this architecture, the neural network first detects the key points of all individuals in the image and then forms part affinity fields describing the affiliation of these points to individual skeletons [28]. Another architecture we utilized was YOLOv8 Pose. Unlike the OpenPose architecture, it belongs to the models with pre-detection of individuals: it first detects individual human figures and then assesses the position of their joints [29]. The YOLO-NAS Pose M architecture performs both human detection and pose estimation in a single step, it enables to reducing the processing time and simplifie calculations without sacrificing accuracy [30]. For comparison completeness, the analysis also included the BlazePose architecture — a lightweight pose estimation model designed for real-time operation on mobile and embedded devices [31]. Based on the analysis of mathematical models, the YOLO-NAS Pose M architecture was chosen as the working model for the research.

To select the architectures, we used a combined criterion, which included both the accuracy of key point detection and the stability of pose recovery over time. The mean average precision (mAP) metric was used as a representative measure of the overall quality of key point detection and localization calculated on the standard COCO Keypoints dataset (a benchmark image database with manually annotated human key points used for evaluating the quality of pose recognition algorithms) [32]. This metric indicates how accurately the model determines the position of human joints compared to the ground-truth annotations: the higher the mAP value, the closer the predicted joint coordinates are to their true positions. It serves as a measure of the overall quality of key point detection and localization. We conducted a comparative analysis of the stability of recovered joint coordinates over time and the frequency of outliers leading to unstable skeleton reconstructions. Based on the results, the YOLO-NAS Pose M architecture demonstrated the lowest variability in coordinates and rare outliers, which, along with a comparable mAP value on COCO Keypoints, determined its selection as the primary working model.

The findings were ***statistically processed*** on a personal computer using the StatSoft Statistica v. 13.0 software package, applying Mann–Whitney test (for comparing independent samples), Wilcoxon (for comparing dependent samples), and Spearman’s correlation coefficient. The data were presented as the median and 25^th^ and 75^th^ percentiles of the median. The differences were considered statistically significant if p< 0.05.

## Results

### Distribution of patients by fall risk and their characteristics

When categorizing patients into fall risk groups using the TUG test, a more relevant method of division was employed rather than the classical threshold of 13.5–14.0 s derived from the studies with small sample sizes [2, 33, 34]. The method is based on the original research published by the developers of this test [26]. The methodology was modified due to its inapplicability to patients with severe mobility impairments, which correspond to absolute functional impairment according to the International Classification of Functioning (ICF). Based on the results of evaluating balance impairments according to ICF, fall risk stratification was performed (Table 2). This method of determining fall risk associated with motor function and dynamic balance has already demonstrated its effectiveness in a previous study [35].

**Table 2:** Stratification of fall risk associated with motor function and mobility based on TUG test

Groups (group characteristics)	Fall risk	ICF codes d410–d429	Test time (s)	Number of patients
Group 1 (complete functional independence)	No fall risk	0 — no difficulties (0–4%)	< 10	66 (36%)
Group 2 (no assistance required for standing up from a chair or using the bathroom; moderate assistance needed for taking a shower; no support required for walking, rare use of a cane; able to walk unaided for a distance of 45 m, can climb stairs independently and safely go outside alone)	Low fall risk	1 — slight difficulties (5–24%)	10–19	107 (57%)
Group 3 (minor assistance may be needed for moving from a chair; rarely requires help with using the bathroom and moderate help for taking a shower; uses a cane for walking, rarely needs supervision (with an assistant); requires minor assistance to walk a distance of 45 m; moderate assistance needed for climbing stairs; more frequent support and help required for going outside, can go out alone, but it is not safe)	High fall risk	2 — moderate difficulties (25–49%)	20–29	10 (5%)
Group 4 (frequently requires help moving from a chair, rarely unable to stand; moderate assistance needed for using the bathroom; often requires help for taking a shower; constant use of support while walking (cane, walker, accompanied by an assistant); often unable to walk a distance of 45 m, assistance required for this distance; frequently needs help with climbing stairs, sometimes unable to do so; always requires support and accompaniment when going outside)	Very high fall risk	3 — heavy difficulties (50–95%)	30 or more	4 (2%)
Group 5 (can only be in a sitting or lying position, cannot stand up or walk even with support)	No fall risk	4 — absolute difficulties (96–100%)	Unable to perform a test	0 (0%)

### Results of patient clustering using machine learning methods and biomechanical analysis, their correlation with fall risk

#### Model for determining step phases

In the first stage, a feature set was calculated for each frame based on cleaned and adjusted data, considering the change in the distance to the camera: joint angles (anatomically corresponding to angles in the joints) and lengths of projections of individual body segments. After that, a set of models was built to determine the step phases. Binary variables corresponding to the timestamps of events such as heel strike, toe strike, heel off, and toe lift for both the right and left legs were used as target variables.

**Figure 1.**
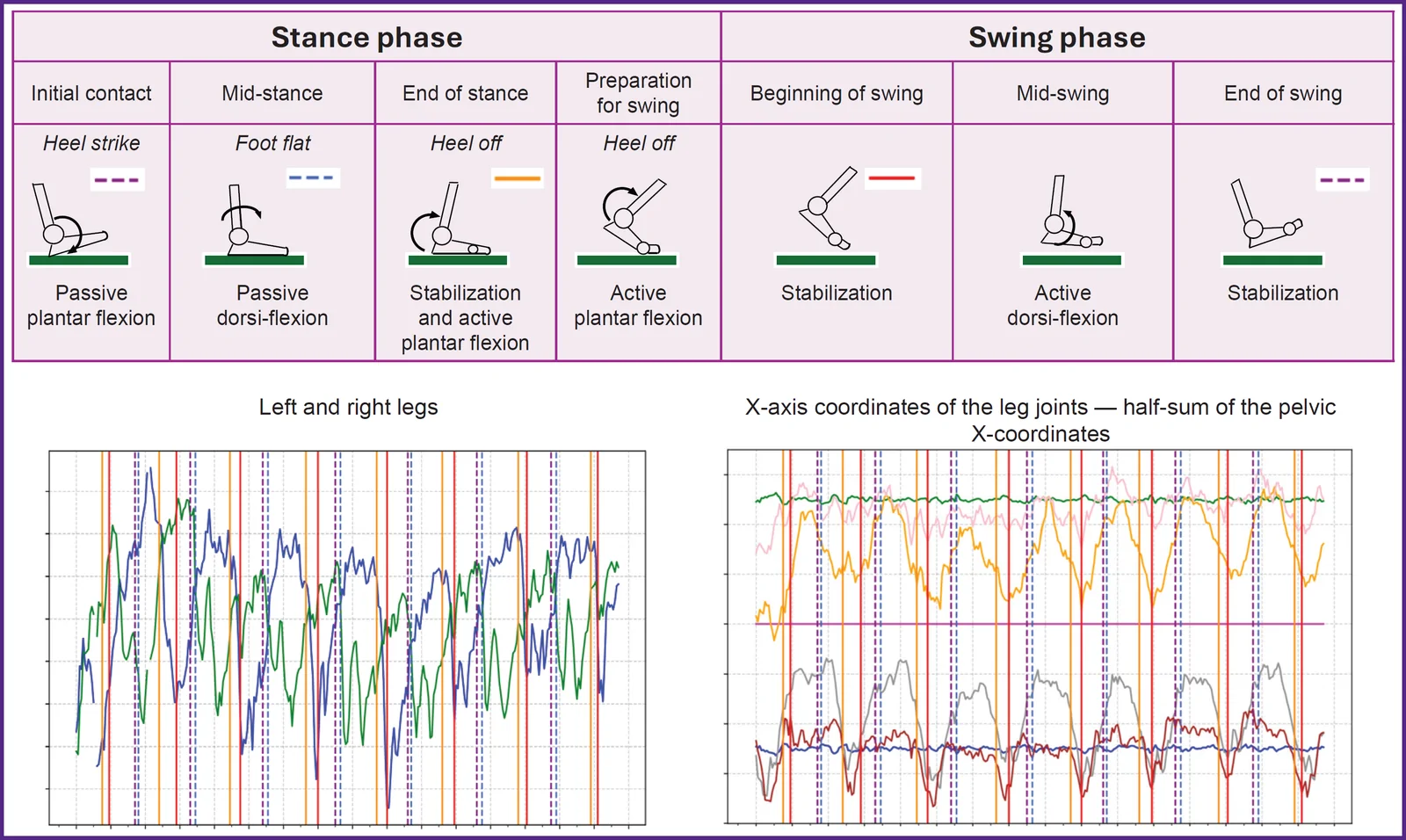
Alignment of AI step phase markings with classical biomechanical phases

To train the model predicting the boundaries of step phases, we performed the manual annotation of 200 gait cycles, with frame numbers marked for toe lift, heel strike, toe strike, and heel off, corresponding to the step phases (Figure 1).

The AI models were provided with a dataset of gait features and event annotations as Figure 1 shows, for the left and right legs, respectively. The data were normalized and cleaned of missing values. For each frame, a context window was formed, which was unfolded into a single vector of features related to the kinematic characteristics of the step (the lengths of projections of individual body segments, joint angles). Machine learning algorithms using the random forest method were applied to these examples in a multi-output setup: a separate binary classifier was trained for each biomechanical event followed by tuning of the algorithm external settings (e.g., learning rate, model depth).

For the unlabeled data, the same parameters were determined, and after processing with the AI model, a list of probability arrays was generated — one for each event (heel strike, toe strike, heel off, and foot lift). Then, the step duration was established, i.e., how many frames pass between two identical gait events.

Initially, the average step period was assessed based on the frequency of movement repetitions; we applied Fourier transformation for both biomechanical signals and neural network responses. It provided several independent estimates of how frequently a step occurs.

Then, these estimates were refined based on actual gait events: the moments of foot contact and lift were identified from the model outputs, and a specific number of frames was selected so that the intervals between these events best corresponded to whole steps. This resulted in more accurate, “event-aligned” estimates of step length.

From all obtained estimates, those that deviated significantly from the majority were removed: first, the median (most typical) step duration was calculated, and then the values that deviated from it by more than 30% were discarded. Subsequently, the extreme 15% of the largest and smallest values were additionally removed to exclude rare outliers.

Next, a stepwise identification of biomechanical events was performed based on the model output probabilities. Initially, we selected the events with high reliability, and as the probability thresholds were lowered, less pronounced candidates were added, but only if they were consistent with the previously assessed step duration and with each other. The regularity of time intervals and the symmetry between the right and left limbs were monitored.

The areas at the beginning and the end of the time series characterized by increased uncertainty were excluded from the analysis, and all events outside the reliable zone were discarded. The correctness of phase order within each step was then checked, and if necessary, the missing events were restored if their temporal position matched the expected step structure and assessed duration.

As a result, a feature set with phase labeling for each video was formed. The results of the phase assessment for each of the AI models are presented in Table 3.

**Table 3:** Separation of step phases (in seconds)

Event	MAE	RMSE
Right leg lift	0.069	0.218
Right heel strike	0.085	0.176
Left toe strike	0.087	0.179
Right heel off	0.104	0.227
Right toe strike	0.085	0.158
Left heel strike	0.085	0.216
Left leg lift	0.078	0.223
Left heel off	0.091	0.216

N o t e. MAE — mean absolute error; RMSE — root mean square error.

#### Selection criteria for steps

Based on the predictions obtained, the steps were excluded from the further sample if the predicted step length differed from the predicted length using Fourier method by more than 20%, and if the stance phase length fell outside the range of 0.3 to 0.7 of the predicted step length. The steps were also excluded where the values of the main features indicated statistical outliers.

#### First-level model

In this model, the analysis of fall risk prediction by the AI system was conducted based on the analysis of a single step.

After selecting quality steps, a feature vector was formed for each step including extreme values (minimums/maximums), joint angle ratios, limb segment lengths, as well as the speeds and accelerations of individual joints. For further analysis, the data were divided into two groups depending on the direction of the passages: toward the camera and away from the camera. At the first stage of modeling, the logarithmically transformed time to complete TUG test was approximated using linear regression based on the normalized step length (step length divided by the framing frequency). For the subsequent error analysis of the linear model and to enhance predictive accuracy, three machine learning models were compared: “decision tree”, “random forest”, and one implementation of the “gradient boosting” model (Table 4) [36].

**Table 4:** Quality metrics for the first-level TUG time prediction model for passages away from the camera (in seconds)

Model	MAE	MSE	RMSE	R^2^
Decision tree	3.092	16.102	4.012	0.403
Random forest	2.680	12.018	3.466	0.554
LGBM (one of gradient boosting implementations)	3.186	17.054	4.129	0.367

N o t e. MAE — mean absolute error; MSE — mean square error; RMSE — root mean square error; R^2^ — R square.

According to the results of the comparative analysis, the “random forest” model demonstrated the highest accuracy. This can be explained by the fact that the algorithm is suitable when a stable model with good generalization ability is needed to work with noisy data. The “random forest” model also showed a lower tendency to overfit compared to the “gradient boosting” model, especially in conditions of limited data volume (Table 5).

**Table 5:** Quality metrics for the first-level TUG time prediction model for passages toward the camera (in seconds)

Model	MAE	MSE	RMSE	R^2^
Decision tree	1.739	5.408	2.326	0.331
Random forest	1.703	4.207	2.051	0.480
LGBM (one of gradient boosting implementations)	1.800	4.696	2.167	0.419

N o t e. MAE — mean absolute error; MSE — mean square error; RMSE — root mean square error; R^2^ — R square.

**Figure 2.**
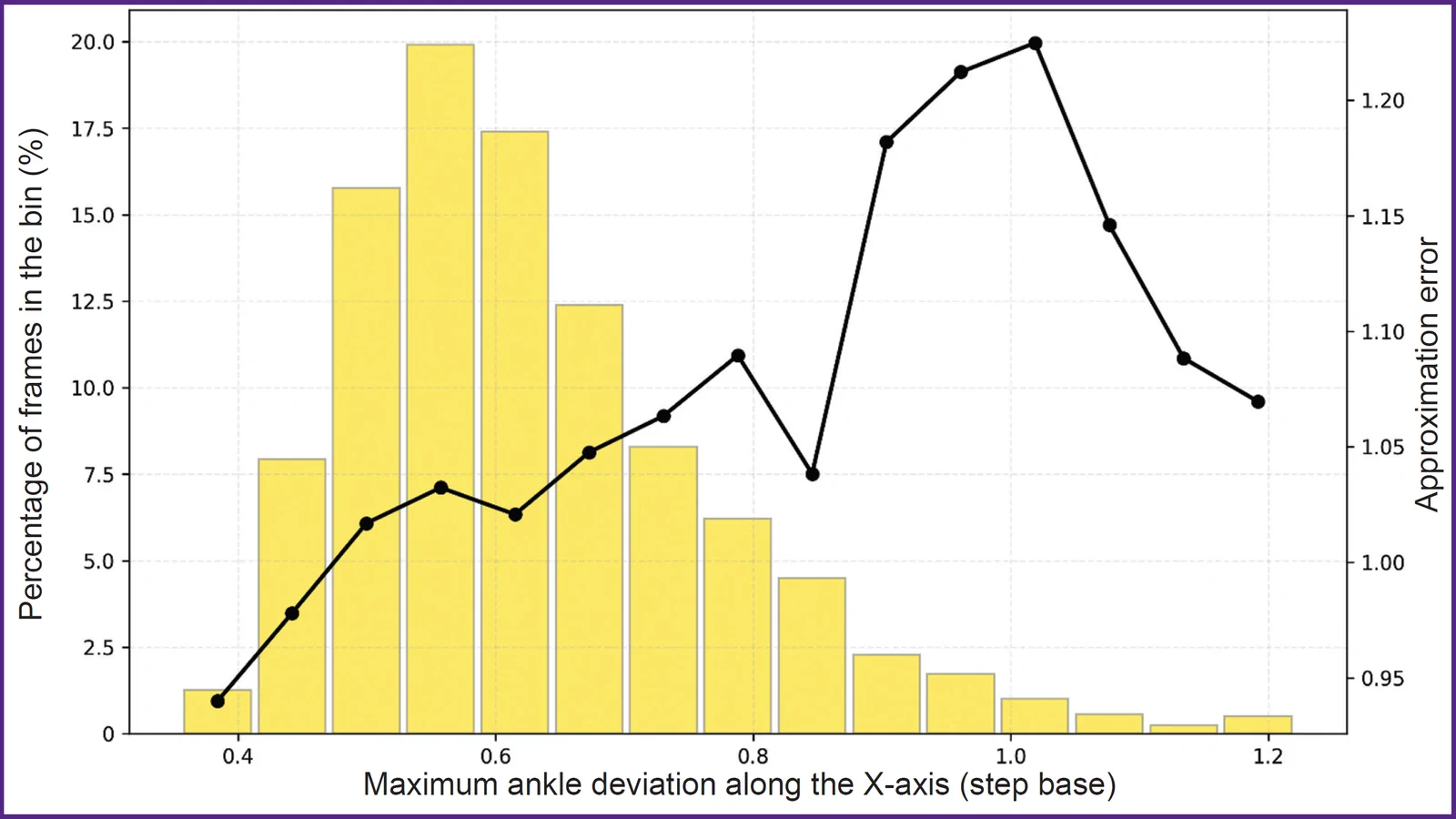
Dependence of TUG time approximation errors on the value of maximum foot deviation along the X-axis (step base) and the distribution of the number of steps by the values of this feature

**Figure 3.**
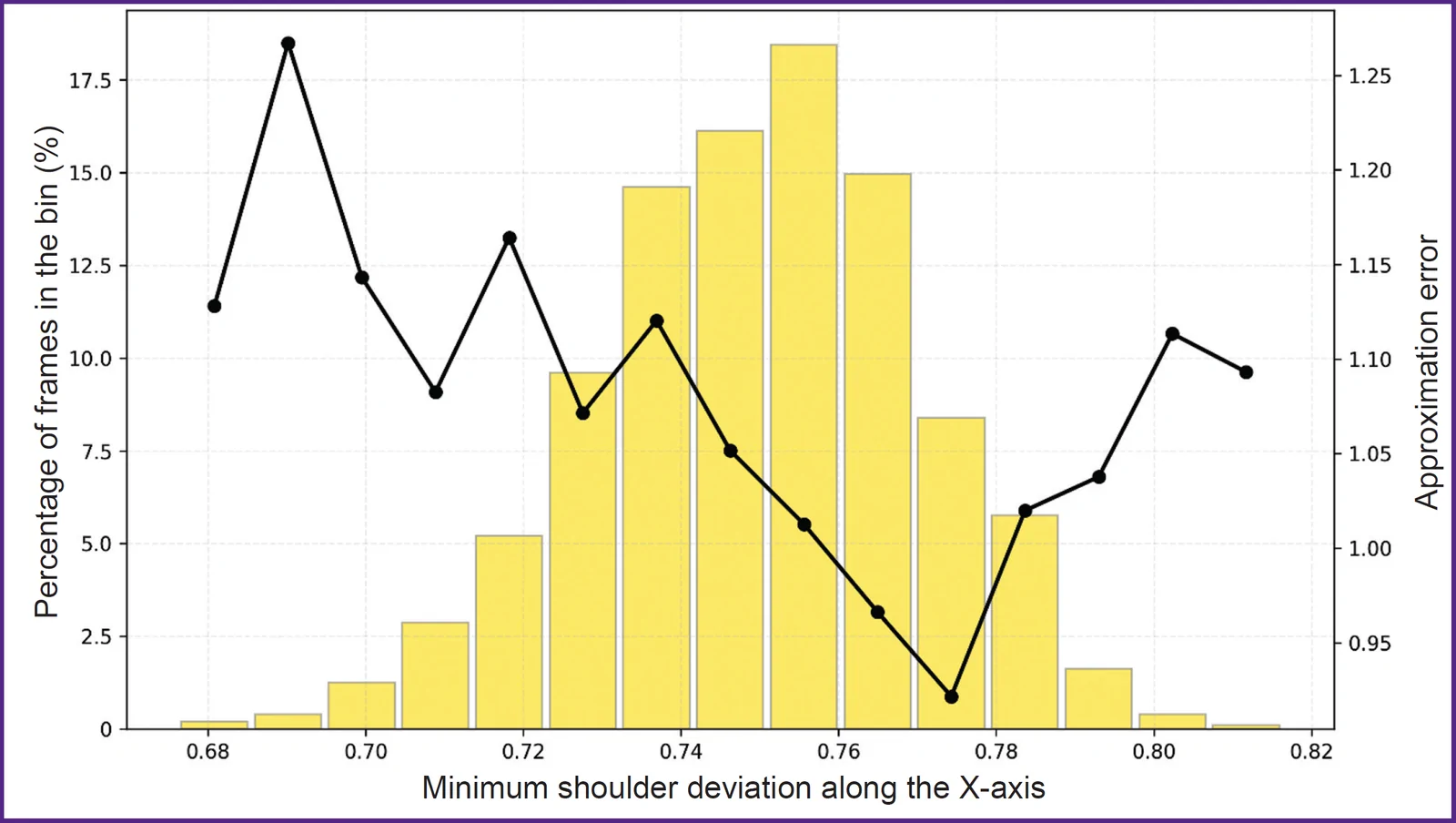
Dependence of TUG time approximation errors on the value of minimum shoulder deviation along the X-axis and the distribution of the number of steps by the values of this feature

**Figure 4.**
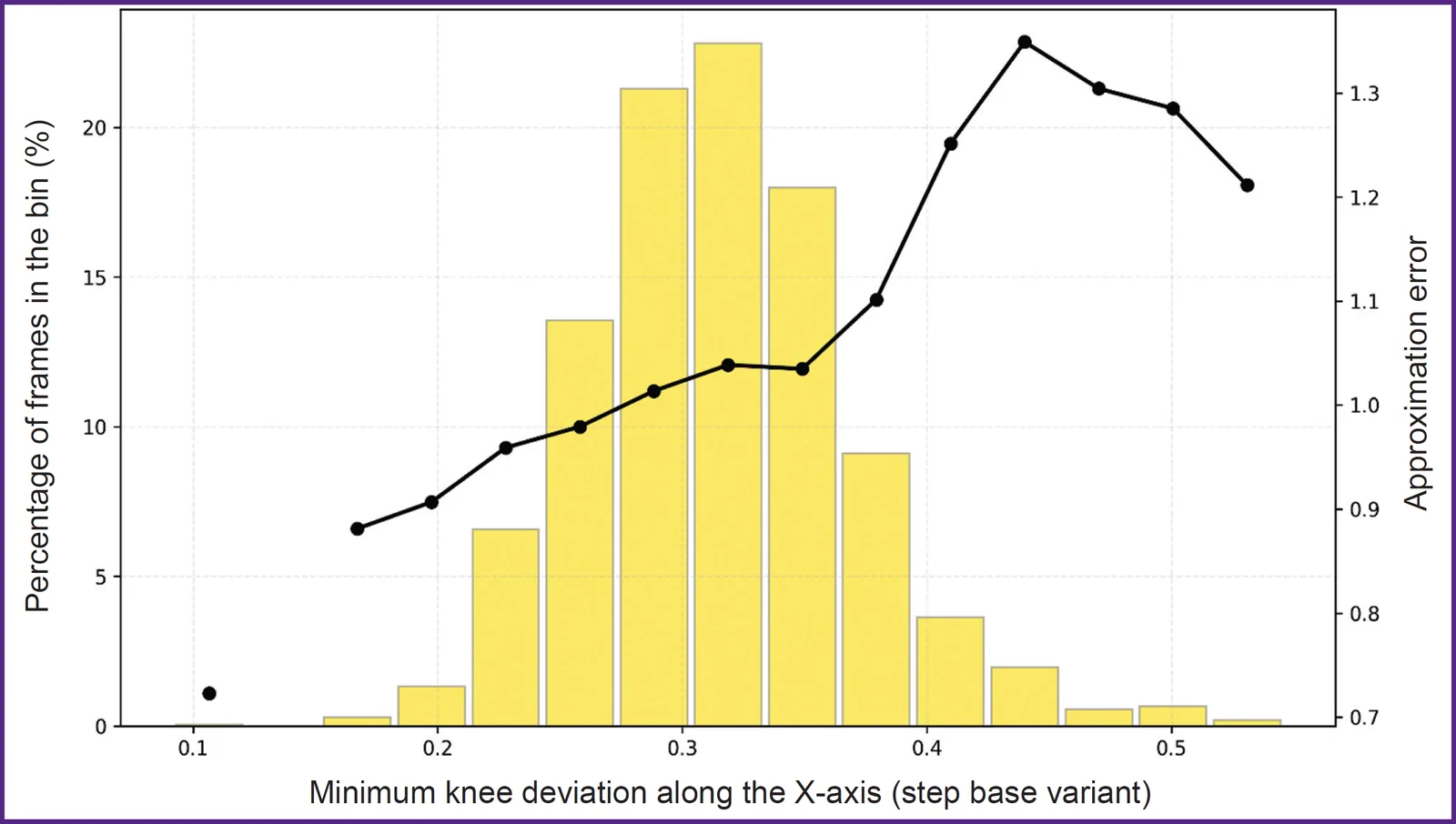
The dependence of TUG time approximation errors on the minimum knee deviation along the X-axis and the distribution of the number of steps based on this feature

**Figure 5.**
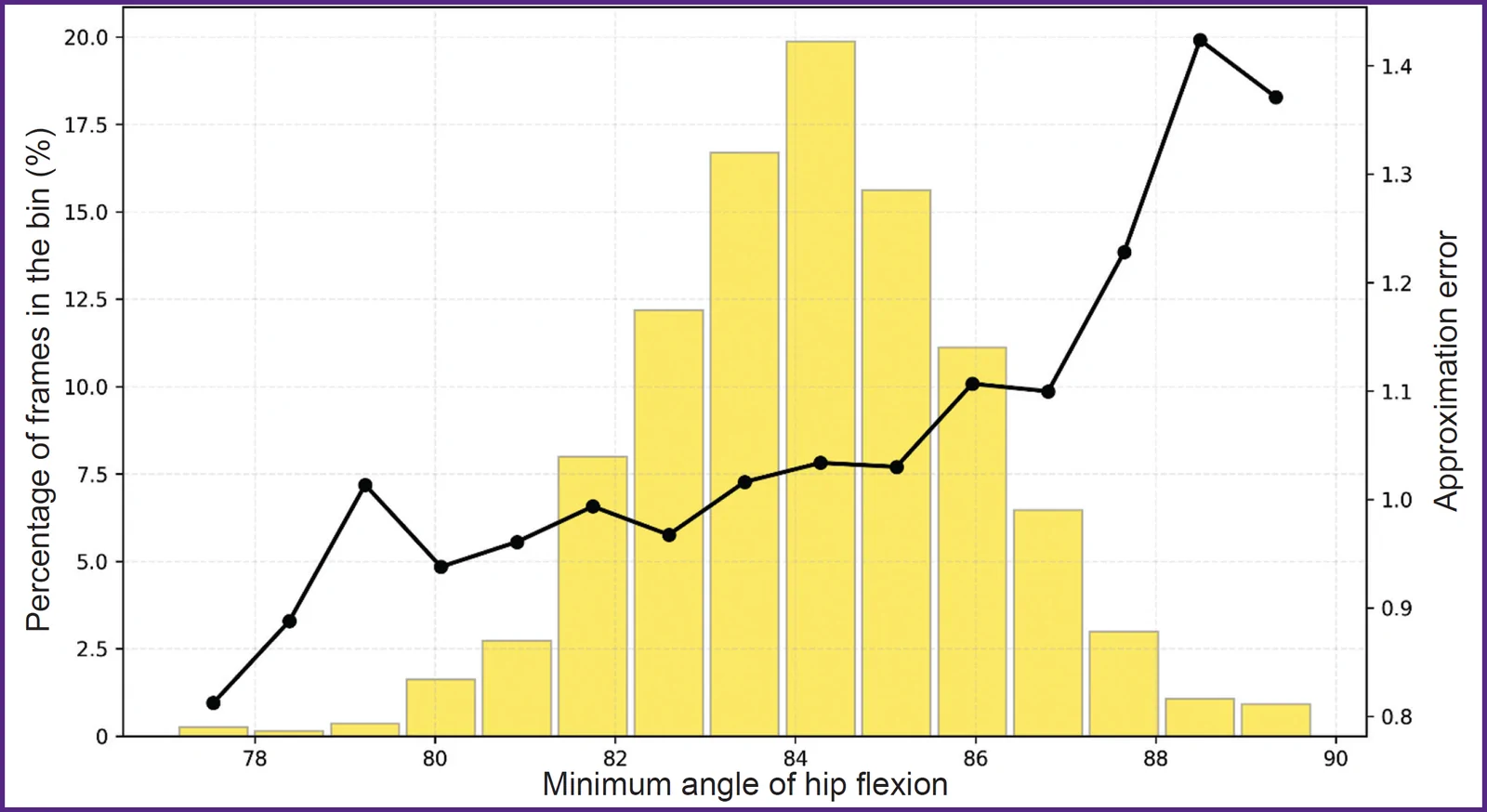
The dependence of TUG time approximation errors on the minimum angle of hip flexion and the distribution of the number of steps based on this feature

**Figure 6.**
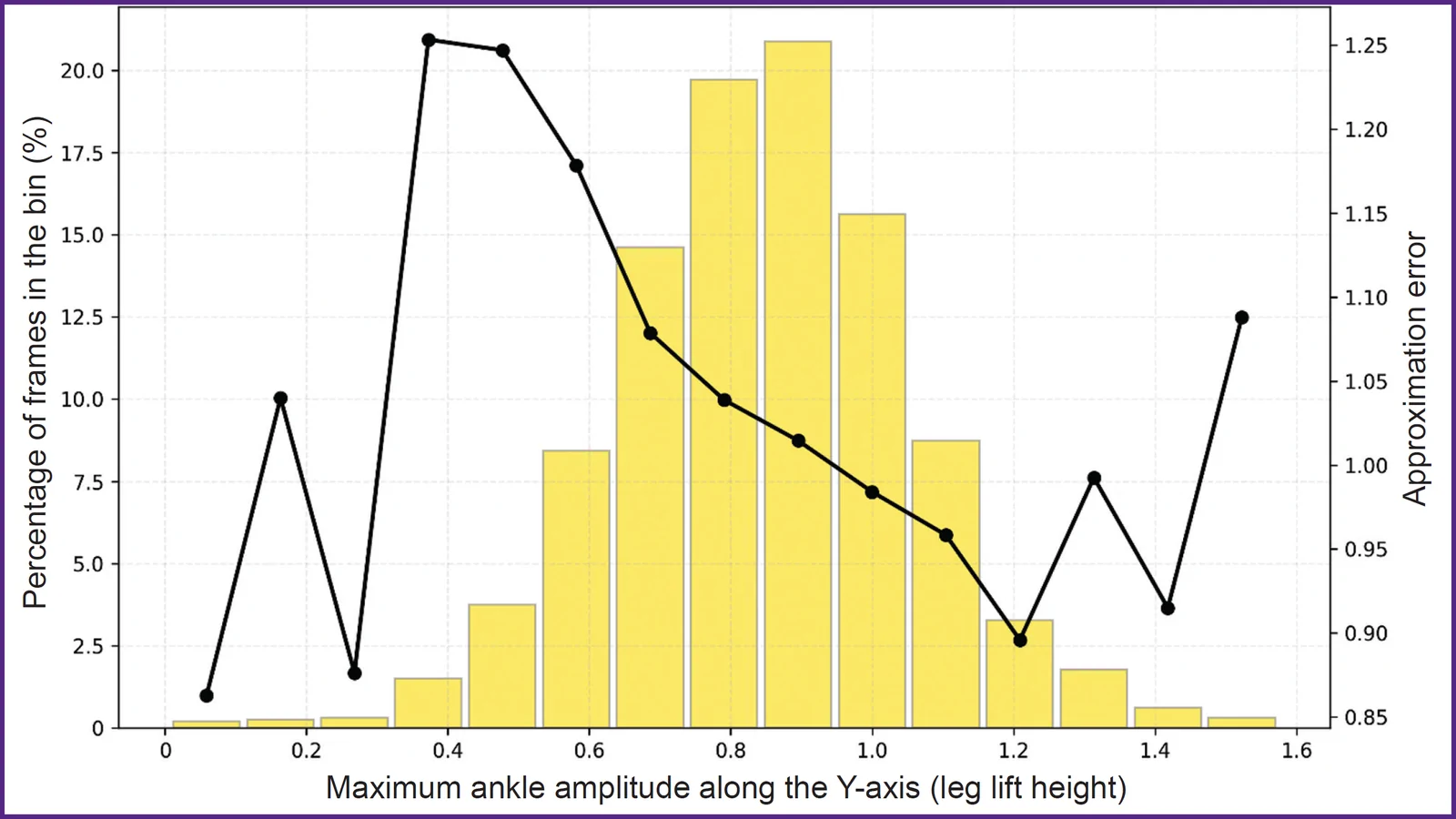
The dependence of TUG time approximation errors on the amplitude of foot lift along the Y-axis and the distribution of the number of steps based on this feature

**Figure 7.**
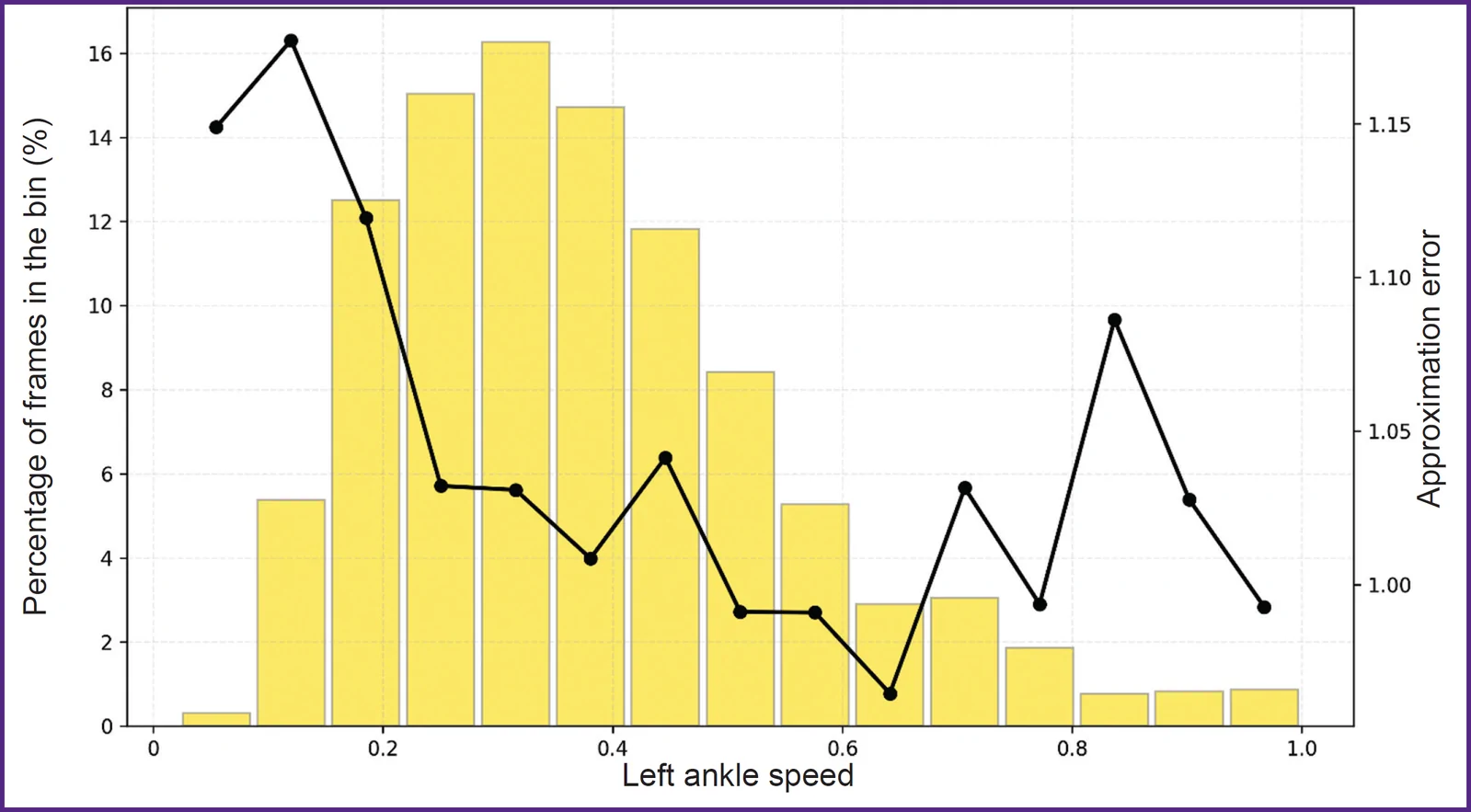
The dependence of TUG time approximation errors on foot movement speed per frame and the distribution of the number of steps based on this feature

Furthermore, based on statistical analysis, we identified the most informative features characterizing the overall stability and safety of gait and which are associated with an increased risk of falling. These features formed the basis for the development of a first-level predictive model:

1. The maximum foot deviation along the X-axis (Figure 2) reflects lateral shifts of the foot: small values were characteristic of high walking speed, which was evident in the video with the foot placement at the same level; excessively large values were characteristic of a wide-based gait (compensation for instability). Thus, this indicator allowed the AI model to predict the risk of falling quite accurately, and the risk linearly increased with the step base indicator.
2. The minimum shoulder deviation along the X-axis (Figure 3) describes lateral body oscillations during walking: an increase in the indicator is associated with a higher probability of losing balance. This indicator significantly increased in our sample with the rising risk of falling, as determined by TUG test, which underscored its diagnostic significance as an additional criterion to assess fall risk.
3. The minimum knee deviation along the X-axis (Figure 4) serves as an indicator of lateral stability of the lower limbs. The indicator was significantly associated with an increased fall risk, and the higher the risk, the greater the variability of this indicator was observed in patients. Overall, this indicator is a variant of calculating the step base, and its changes significantly correlated with the minimum ankle joint deviation along the X-axis.
4. The minimum angle of hip flexion (Figure 5) is associated with the functional “range of motion”: low values for this indicator are accompanied by a shortened stride due to insufficient forward leg extension and a decrease in walking speed, which limits the ability to react quickly to obstacles. In patients, an increase in the fall risk measured by TUG scale was observed to correlate with a decrease in the indicator.
5. The amplitude of foot lift along the Y-axis (Figure 6) characterizes the vertical clearance of the foot: in patients from our sample, insufficient foot lift increased the risk of tripping, while excessive lift often reflected an energy-consuming, inefficient walking pattern. Both conditions were associated with an increased fall risk in patients.
6. The speed of foot movement frame by frame (Figure 7) reflects the pace and smoothness of the step. Low speed indicates uncertainty or weakness, while sharp fluctuations indicate gait instability. In patients from our sample, a significant decrease in foot movement speed was noted with an increased fall risk according to TUG.

Together, these indicators provide a comprehensive picture of dynamic balance, walking biomechanics, and “step safety” helping to more accurately assess the risk of falling.

When interpreting the quality metrics of the AI model, it is essential to consider the error of the target variable. Clinically, the time taken to perform TUG test was recorded manually, and it could lead to overestimations due to observer reaction and delays in starting/stopping (no systematic research on possible errors was conducted, but in some cases, deviations of up to 2 s were observed). It introduced additional noise and bias, inflated the MAE/MSE values, reduced R^2^, and distorted the comparative assessment of the models.

#### Second-level model

To generate predictions for fall risk using the AI system for each video, unlike the level one model, where the unit of observation was a single step, a dataset was created based on aggregating step-by-step predictions from the level one model and subsequent regression of the target variable.

For each recording, a feature vector was formed including the characteristics of the level one model predictions (minimum, maximum, arithmetic mean, median, lower quartile, upper quartile, measures of dispersion and distribution shape — standard deviation, coefficient of variation, interquartile range, median absolute deviation, range, as well as skewness and kurtosis of TUG prediction time obtained for each step within the video); the variances across a set of pre-selected biomechanical characteristics (these characteristics included maximum/minimum deviation of the foot, knee, and shoulder along the X-axis, foot movement speed, minimum angle of hip flexion, and foot lift amplitude); and normalized step length. Such dataset allowed the final model to consider not only the actual level one predictions for each step in the video but also their stability/heterogeneity within the video, which enhanced resilience to outliers.

Then, the data was split into training and testing samples, and a feature matrix was formed by excluding non-informative identifiers and auxiliary fields. Various algorithms were compared as regression approximators at the second level (“random forest”, “decision tree”, and one of the implementations of “gradient boosting”). The quality was assessed on a holdout sample using MAE, RMSE, and the coefficient of determination R^2^. The result was a level two model reflecting the structure of averaged and variable characteristics of the level-one predictions and the associated biomechanical parameters providing a more reliable and interpretable assessment compared to using individual steps.

The results of the second-level model evaluation showed that for the passages away from the camera, the “random forest” model exhibited the lowest error (Table 6). For the passages to the camera, the LGBM model showed a slightly lower error compared to the “random forest” (Table 7); however, the difference between the models was insignificant. Meanwhile, the “random forest”, as in the previous stage (see Tables 4 and 5), demonstrated more stable results and a lower tendency to overfit with limited data. Considering this and to maintain a unified architecture for the final model for both shooting directions, the “random forest” was chosen as the working second-level model.

**Table 6:** Quality metrics for the second-level TUG time prediction model for passages away from the camera (in seconds)

Model	MAE	MSE	RMSE	R^2^
Decision tree	2.078	6.531	2.556	0.721
Random forest	1.835	5.297	2.302	0.774
LGBM (one of gradient boosting implementations)	2.601	12.118	3.481	0.482

N o t e. MAE — mean absolute error; MSE — mean square error; RMSE — root mean square error; R^2^ — R square.

**Table 7:** Quality metrics for the second-level TUG time prediction model for passages toward the camera (in seconds)

Model	MAE	MSE	RMSE	R^2^
Decision tree	2.262	10.223	3.197	–0.358
Random forest	1.445	3.634	1.906	0.517
LGBM (one of gradient boosting implementations)	1.418	3.054	1.748	0.594

N o t e. MAE — mean absolute error; MSE — mean square error; RMSE — root mean square error; R^2^ — R square.

#### Results of group determination

Additionally, we performed the stratification of patient video recordings into groups based on fall risk levels (Table 8) based on the time taken to complete TUG test for a visual verification of the quality of group distribution according to Table 2 [35].

**Table 8:** The accuracy of predictions into actual fall risk groups

Artificial intelligence-predicted group	Actual group according to TUG			
	No risk	Low risk	High risk	Very high risk
No risk	90*	58**	0	0
Low risk	31**	202*	2**	0
High risk	0	6**	18*	0
Very high risk	0	0	2**	2*

N o t e. The asterisks indicate the accuracy of artificial intelligence classification relative to the reference TUG test:

*corresponds to correct fall risk recognition (number of cases correctly identified),

** indicates false prediction.

## Discussion

As a clinical model for fall risk screening of the studied patient cohort, we selected TUG test as the simplest and most accurate method for quantitatively assessing the motor components of fall risk [7] in patients with neurological movement disorders [37]. The test has already been used to train AI to predict fall risk, although only the recording of TUG test itself was analyzed [38]. In developing our model, we focused on analyzing the patients’ walking in a free mode along a corridor, without additional conditions (i.e., in conditions closest to real practice in outpatient and inpatient settings). The AI model developed as a result of the present study was able to determine fall risk with 76% accuracy (similar to TUG) based on a simple walk of the patient toward or away from the camera (see Table 8). In a previously published study by Angsuwan et al. [39], the sensitivity of the random forest model for determining fall risk based on TUG using biomechanical data obtained through accelerometry was higher compared to our data: the AUC (area under the ROC curve) was 0.98 indicating a high accuracy of fall risk prediction. In our work, the average absolute error in the best prediction model during walking toward the camera was 1.445 s, which may be attributed, firstly, to manual input error, i.e., the speed of the researcher’s button press on the stopwatch (This was confirmed by the fact that for some patients, the speed of performing the test measured by video and manually, significantly differed). Secondly, we used a different grading of fall risk than other researchers, based on the assessment of the patient functional capabilities and dividing them into 5 risk groups instead of 2, which provides a more comprehensive and accurate assessment of fall risks related to motor function. However, a greater number of risk gradations require more precise measurements and predictive capability.

Based on the manual annotation of gait phases we conducted, we developed an algorithm for automatic gait phase annotation using an AI system. It is worth noting that the phases annotated by the AI system show fewer events in the stance and swing phases compared to the classical representations of walking biomechanics [40]. It is due to the specifics of how the patient video is recorded (either from the camera or towards the camera), which prevents from complete identification of all basic events in the gait phases compared to side-angle filming. However, considering user convenience, the filming format we suggested is preferable. It should be noted that the detection of the main phases (stance and swing) is accurate, which is sufficient for the primary aim of the research: developing an algorithm for predicting fall risk based on smartphone camera recordings.

The data obtained from the AI system processing enabled us to identify the main biomechanical predictors of fall risk: step base (maximum ankle deviation along the X-axis), lateral body sway (minimum shoulder deviation along the X-axis), step base at the knee joint level (minimum knee deviation along the X-axis), vertical foot clearance (foot lift amplitude), and step speed (foot movement speed). Our findings are consistent with published data: when using wearable sensors (accelerometric motion analysis systems), the key biomechanical factors that help identify patients prone to falls also include step speed, foot lift clearance, and step variability [41]. Furthermore, as a result of the AI assessment of the biomechanical model of whole-body movements during walking, we identified additional factors significant for fall risk assessment: lateral body sway and step base. It is an advantage of evaluating the complete video recording of walking compared to the assessments using accelerometric sensors, which mainly evaluate movements of the lower limbs.

Fall risk prediction classification models based on the analysis of the patient general status (clinical and socio-demographic factors, medication use, etc.) have an accuracy of around 78% when applied to a neurological patient population [42]. In turn, the models based on a comprehensive analysis of general status and instrumental assessment of gait changes, increase prediction accuracy to 92% [42]. In the present study, we focused on analyzing biomechanical indicators and assessing the relationship between fall risk, mobility, and gait disturbances — a leading factor in balance disorders among neurological patients. In the future, to enhance the accuracy of fall risk prediction, we are planning to conduct additional analyses of the role of other risk factors and their relationships with identified motor disturbances, which may contribute to improving the quality of the developed classifier.

Despite the development of methods for analyzing gait video recordings using neural networks and computer vision, without the application of marker systems and deep learning models for fall risk assessment [43, 44], most existing studies still focus either on a limited set of temporal gait characteristics or on analyzing short segments of movement.

In the present study, the following assumptions are made:

the errors in key point extraction are small and do not lead to systematic bias;
filtering and removing rare quantiles are sufficient to suppress artifacts;
frame rate, angle, lighting, and distance to the camera are comparable across recordings;
domain shift is minimal; only 2D coordinates obtained from a single camera are used, with depth and vertical components of movement assessed through indirect features; the time taken to complete TUG test is considered a reliable benchmark, despite potential systematic overestimation related to both manual timing recording and inter-observer variability;
steps within a single recording are aggregated as conditionally independent observations;
sampling by patient indices (rather than by individual steps or recordings) prevents data leakage between training and testing sets — otherwise, the model might “memorize” individual patient characteristics rather than identifying general disease patterns.

A strength of our study is the comprehensive biomechanical analysis of gait in neurological patients using a two-level AI model, which enables to consider both individual step parameters and movement variability across the entire walking video recording of the subject — both towards and away from the camera.

### Study limitations

Nearly half of the recorded steps were excluded during data adjustment. Some exclusions were due to significant inaccuracies in registering the start and end of the gait. Others were related to the errors made by the neural network in placing anthropometric points, as well as the errors in detecting gait phases. This portion of errors will be the focus in further improving the model.

## Conclusion

As a result of our clinical-biomechanical study, we developed an AI model that can predict the fall risk in neurological patients with 76% accuracy. This model is based on an objective assessment method — video recording of walking followed by mathematical and biomechanical analysis of movement patterns. In our future work, we will consider additional fall risk factors (history of falls, use of antidepressants, and other medications) to improve prediction accuracy, including general clinical characteristics of the disease. Among the potential improvements for the mathematical processing of walking video data, we may explore the application of cross-validation grouped by patients, the use of stricter smoothing and trajectory recovery methods, the implementation of neural network architectures (Temporal CNN, LSTM/GRU, Transformer), as well as other machine learning methods, such as ensembling, with an increased dataset (number of patients).
